# Dual-modality imaging with CEUS and MRI for early recurrence assessment in hepatocellular carcinoma following combined radiofrequency ablation and transarterial chemoembolization

**DOI:** 10.3389/fonc.2026.1854471

**Published:** 2026-09-04

**Authors:** Shuqiang Jin, Qimeihui Wang, Lingyu Zhu, Dengke Teng, Guoqing Sui, Hui Wang

**Affiliations:** Department of Ultrasound, China-Japan Union Hospital of Jilin University, Changchun, Jilin, China

**Keywords:** contrast-enhanced ultrasound (CEUS), early recurrence diagnosis, hepatocellular carcinoma (HCC), magnetic resonance imaging (MRI), radiofrequency ablation (RFA), transarterial chemoembolization (TACE)

## Abstract

**Objective:**

This study aimed to evaluate the diagnostic performance of combined contrast-enhanced ultrasound (CEUS) and magnetic resonance imaging (MRI) for detecting early recurrence within 12 months after radiofrequency ablation combined with transarterial chemoembolization (RFA-TACE) in patients with hepatocellular carcinoma (HCC).

**Methods:**

This retrospective cohort study included 173 patients with HCC who underwent RFA-TACE between July 2021 and July 2023, including 143 men and 30 women, with a mean age of 57.35 ± 7.61 years. All patients underwent CEUS and 3.0-T MRI at 1 and 3 months after RFA-TACE, with additional examinations performed when recurrence was subsequently suspected. Two senior radiologists independently reviewed the images while blinded to the clinical outcomes. CEUS assessment included a whole-liver grayscale survey followed by targeted contrast-enhanced evaluation of the treated area and any additional suspicious intrahepatic lesions. MRI assessment included whole-liver evaluation of qualitative enhancement and diffusion characteristics together with quantitative apparent diffusion coefficient values and ratios. Early recurrence was assessed using CEUS-only interpretation, MRI-only interpretation, and an exploratory combined CEUS–MRI interpretation strategy. The combined strategy represented expert integrated judgment rather than a prespecified algorithmic model. Disagreements were resolved by a third senior radiologist. Recurrence status was determined using a composite reference standard based on histopathology when available or, otherwise, longitudinal clinical and imaging follow-up adjudicated independently of the index-test interpretations. Diagnostic performance was summarized using sensitivity, specificity, positive predictive value, negative predictive value, and accuracy.

**Results:**

Early recurrence occurred in 109 of 173 patients (63.01%). In unadjusted comparisons, the early recurrence group had higher proportions of BCLC stage C disease (48.62% vs 18.75%; OR = 4.10, 95% CI: 1.97–8.52; P<0.001), Child-Pugh class B liver function (32.11% vs 12.50%; OR = 3.31, 95% CI: 1.43–7.69; P = 0.004), and pretreatment AFP levels >100 ng/mL (59.63% vs 42.19%; OR = 2.02, 95% CI: 1.08–3.79; P = 0.026). Tumor diameter >5 cm was not significantly associated with early recurrence (45.87% vs 31.25%; OR = 1.86, 95% CI: 0.97–3.57; P = 0.059). On CEUS, arterial-phase nodular or irregular enhancement, early contrast washout, peritumoral enhancement, and irregular margins were more frequent in the early recurrence group (all P ≤ 0.001). On MRI, DWI hyperintensity, arterial-phase nodular or irregular enhancement, delayed-phase washout, and irregular margins were also more frequent in the early recurrence group. The mean ADC value was lower in the early recurrence group than in the no early recurrence group (1.05 ± 0.18 vs 1.32 ± 0.15 ×10^-3^ mm²/s, P<0.001). CEUS alone achieved a sensitivity of 80.7%, specificity of 82.8%, and accuracy of 81.5%. The corresponding values were 88.1%, 87.5%, and 87.9% for MRI and 92.7%, 93.8%, and 93.1% for combined CEUS–MRI.

**Conclusion:**

Exploratory combined CEUS–MRI interpretation showed higher descriptive sensitivity, specificity, and accuracy than either CEUS-only or MRI-only interpretation for detecting early recurrence after RFA-TACE. CEUS and MRI provided complementary information on vascular perfusion, tissue structure, and water-molecule diffusion. Because the combined strategy was based on expert integrated judgment rather than a prespecified algorithm, its findings should be interpreted cautiously and require prospective validation.

## 1 Introduction

Hepatocellular carcinoma (HCC), the predominant histological subtype of primary liver cancer, remains a major global health burden. In 2022, approximately 865,000 new liver cancer cases and 758,000 liver cancer-related deaths were estimated worldwide, making liver cancer the sixth most frequently diagnosed malignancy and the third leading cause of cancer-related mortality (1). Although hepatic resection, liver transplantation, and local ablation may provide curative-intent treatment for selected patients, their applicability is limited by tumor burden and location, hepatic functional reserve, portal hypertension, comorbidities, donor availability, and the feasibility of achieving an adequate ablation margin (2). Among appropriately selected patients, RFA combined with TACE may be undertaken with curative intent rather than being regarded solely as palliative treatment. A meta-analysis showed that TACE plus RFA achieved overall survival outcomes comparable to those of surgical resection and was associated with lower morbidity; in propensity score-matched analyses, the differences in 3- and 5-year disease-free survival were not statistically significant (3). Additional comparative evidence has shown that TACE-RFA can achieve acceptable local tumor control in selected HCCs that are not feasible for conventional ultrasound-guided RFA (4). Nevertheless, early post-treatment recurrence after RFA-TACE remains a major obstacle to durable disease control. In the present study, early recurrence was defined as viable HCC detected within 12 months after completion of RFA-TACE, including local tumor progression at or adjacent to the treated area and new intrahepatic recurrence arising elsewhere in the liver. The 12-month cutoff was adopted because previous multicenter evidence identified this interval as a clinically meaningful threshold for distinguishing early from late recurrence based on differences in post-recurrence survival (5). Timely detection is therefore important for selecting subsequent treatment and avoiding missed therapeutic opportunities. Although contrast-enhanced CT, CEUS, and MRI are widely used for post-treatment surveillance (6–8), necrosis, hemorrhage, inflammation, and fibrosis within or surrounding the treated area may complicate image interpretation. CEUS may miss deeply located lesions or lesions in sonographically inaccessible areas, whereas post-treatment inflammation, hemorrhage, necrosis, and fibrosis may complicate CT or MRI interpretation (9–11).

CEUS uses intravascular microbubble contrast agents to provide continuous real-time assessment of microvascular perfusion. Nodular or irregular arterial-phase hyperenhancement followed by contrast washout may indicate viable tumor tissue within or adjacent to the treated area (11–13). Dynamic contrast-enhanced MRI characterizes lesion vascularity across the arterial, portal venous, and delayed phases, while diffusion-weighted imaging and apparent diffusion coefficient measurements reflect water-molecule diffusion; viable hypercellular tissue generally demonstrates restricted diffusion and relatively low ADC values (14). Thus, CEUS provides high-temporal-resolution information on local perfusion, whereas MRI offers whole-liver, multiparametric assessment of vascular enhancement, tissue structure, and diffusion. Although the complementary use of MRI and CEUS in post-treatment surveillance has been explored (6), evidence regarding an integrated dual-modality approach specifically for assessing early recurrence after RFA-TACE remains limited.

This retrospective cohort study aimed to evaluate the diagnostic value of combined CEUS and MRI and to explore the associations between key imaging characteristics and early recurrence, thereby providing evidence for optimizing noninvasive post-treatment follow-up after RFA-TACE.

## 2 Materials and methods

### 2.1 Ethical statement

This retrospective cohort study involved human participants and was approved by the Institutional Ethics Committee of China-Japan Union Hospital, Jilin University [(2025) Clinical Trial Review No. (2025021904)]. The study was conducted in accordance with the Declaration of Helsinki, as revised in 2013. Because the study involved the retrospective analysis of previously collected clinical and imaging data, the requirement for individual informed consent for study participation was waived by the Ethics Committee. All patient data were anonymized and de-identified before analysis. Written informed consent for publication of the detailed clinical and imaging information presented in the representative case was obtained from the patient.

### 2.2 Study design and patient characteristics

This retrospective cohort study included patients with hepatocellular carcinoma (HCC) who underwent combined radiofrequency ablation and transarterial chemoembolization (RFA-TACE) at our institution between July 2021 and July 2023. Baseline demographic and clinical data were retrospectively obtained from the hospital medical record system before RFA-TACE, including age, sex, HCC etiology, Barcelona Clinic Liver Cancer (BCLC) stage, Child-Pugh class, pretreatment alpha-fetoprotein (AFP) level, and tumor diameter.

A total of 173 eligible patients were included, comprising 143 men (82.66%) and 30 women (17.34%). The patients were aged 32–78 years, with a mean age of 57.35 ± 7.61 years. Among them, 108 patients (62.43%) had BCLC stage B disease and 65 (37.57%) had BCLC stage C disease. Child-Pugh class A and B liver function were present in 130 patients (75.14%) and 43 patients (24.86%), respectively. The underlying etiology was hepatitis B virus infection in 127 patients (73.41%), hepatitis C virus infection in 25 patients (14.45%), and other causes in 21 patients (12.14%). Pretreatment AFP levels were >100 ng/mL in 92 patients (53.18%), and 70 patients (40.46%) had a tumor diameter >5 cm.

All patients underwent standardized clinical and imaging follow-up after RFA-TACE. According to recurrence status within 12 months after RFA-TACE, 109 patients were assigned to the early recurrence group and 64 patients to the no early recurrence group. All patient data were anonymized and de-identified before analysis.

### 2.3 Inclusion and exclusion criteria

*Inclusion criteria:*

1. Pathologically confirmed or clinically diagnosed HCC (based on imaging and laboratory tests);
2. Underwent combined RFA-TACE treatment (RFA performed after TACE);
3. BCLC stage B or C;
4. Child-Pugh class A or B liver function;
5. Patients in generally good condition, with stable vital signs, no severe bleeding tendency or active infection, no massive ascites, no hepatic or renal failure, and normal or mildly abnormal coagulation function (correctable to ≥70% of normal values through medical treatment);
6. No contraindications to TACE or RFA;
7. Underwent both CEUS and contrast-enhanced MRI at 1 and 3 months after RFA-TACE, with additional examinations performed at the first subsequent clinical or radiological suspicion of recurrence, and had complete and analyzable imaging data;
8. Complete and standardized post-treatment follow-up records were available, including contrast-enhanced imaging performed during the 12-month follow-up period, allowing recurrence status to be determined using the prespecified composite reference standard.

*Exclusion criteria:*

1. BCLC stage A (more suitable for curative treatment);
2. Child-Pugh class C liver function;
3. Presence of severe comorbidities (e.g., uncontrolled severe diabetes, heart disease, chronic obstructive pulmonary disease, systemic infections);
4. Severe coagulopathy with uncorrectable bleeding tendency (coagulation function not correctable to ≥70% of normal through medical treatment);
5. History of prior treatment for the target lesion (e.g., surgical resection, other ablation techniques, radiotherapy, targeted therapy);
6. Allergy to the contrast agents used (gadopentetate dimeglumine or SonoVue);
7. Failure to complete CEUS and MRI follow-up as required post-RFA-TACE, or incomplete/poor-quality imaging data unsuitable for evaluation;
8. Incomplete follow-up records making it impossible to determine early recurrence status.
9. Persistent viable tumor identified at the initial 1-month post-treatment assessment, indicating incomplete local treatment rather than post-treatment recurrence.

### 2.4 Treatment method (RFA-TACE)

All enrolled patients underwent standardized RFA-TACE combination therapy. The procedure began with TACE, performed using the Seldinger technique to puncture the right femoral artery and insert a catheter, which was super-selectively advanced to the tumor-feeding hepatic artery. Angiography was then used to confirm tumor staining, vascular abnormalities, and blood supply. Based on tumor size, number, and the patient’s liver function status, a chemoembolization emulsion consisting of oxaliplatin (150 mg), pirarubicin (30 mg), and iodized oil (5–20 mL) was slowly injected.

Within an appropriate time window following TACE (typically 1–2 weeks), CT-guided radiofrequency ablation was performed. The tumor was precisely localized, and a safe puncture route was planned. Ablation parameters (including power and duration) were set according to the tumor’s size and location, ensuring that the ablation covered the entire tumor and extended approximately 1 cm beyond its margin into surrounding normal liver tissue. After ablation, patients received appropriate supportive care (e.g., hepatoprotective and analgesic treatment) to facilitate recovery.

### 2.5 CEUS and MRI examination protocols

All patients underwent CEUS and contrast-enhanced MRI at 1 and 3 months after RFA-TACE. When recurrence was clinically or radiologically suspected during subsequent follow-up, both examinations were repeated to ensure consistent multimodal evaluation.

MRI examinations were performed using a 3.0-T scanner (Signa HDX, GE Healthcare). The liver MRI protocol included T1-weighted imaging, T2-weighted imaging, in-phase and opposed-phase T1-weighted imaging, diffusion-weighted imaging with a b-value of 1000 s/mm², and apparent diffusion coefficient mapping. For dynamic contrast-enhanced MRI, gadopentetate dimeglumine was administered intravenously through an antecubital vein at a dose of 0.2 mL/kg and an injection rate of 2 mL/s. Bolus tracking was used to initiate dynamic acquisition. Arterial-phase images were acquired 25–30 seconds after contrast administration, portal venous-phase images at 55–65 seconds and delayed-phase images at 2–3 minutes. The entire liver was systematically evaluated throughout the three dynamic phases, including the treated area, its margins, and the remaining liver parenchyma, to identify both local tumor progression and newly developed intrahepatic lesions.

CEUS examinations were performed using an EPIQ 7C ultrasound system (Philips Healthcare) equipped with a C251 convex-array transducer operating at 2.0–5.0 MHz. Patients fasted for 12 hours before the examination. Conventional grayscale ultrasound was first used to survey the entire liver and to assess the location, morphology, size, echogenicity, and margins of the treated area. Particular attention was paid to the treated-area margin and to any newly detected focal lesion elsewhere in the liver. The system was then switched to contrast-specific imaging mode. A 2.4-mL bolus of SonoVue was rapidly administered through an antecubital vein over 2–3 seconds, followed by a 5-mL saline flush, and the timer was started simultaneously. The treated area and adjacent liver tissue were continuously observed for at least 360 seconds. CEUS phases were defined as the arterial phase at 10–30 seconds, the portal venous phase at 30–120 seconds, and the delayed phase at 120–360 seconds. Enhancement morphology, time of initial enhancement, time to peak enhancement, and the presence, timing, and speed of contrast washout were recorded. CEUS was primarily targeted to the treated area; when an additional suspicious intrahepatic lesion was identified during the whole-liver survey, targeted contrast-enhanced evaluation of that lesion was also performed.

### 2.6 Imaging evaluation indicators and assessment workflow

The diagnostic performance of CEUS and contrast-enhanced MRI was evaluated at the patient level using examinations performed at 1 and 3 months after RFA-TACE or at the first subsequent clinical or radiological suspicion of recurrence. The final assessment included both the treated area and the remaining liver parenchyma. Early post-treatment recurrence was defined as viable HCC detected within 12 months after RFA-TACE after complete response had been documented at the initial post-treatment assessment. Local tumor progression was defined as newly developed viable tumor at or adjacent to the treated area after at least one previous contrast-enhanced examination had demonstrated complete response. New intrahepatic recurrence was defined as a newly developed HCC lesion arising elsewhere in the liver, spatially separate from the treated area. Persistent viable enhancement identified at the initial 1-month assessment was classified as residual viable tumor or incomplete local treatment rather than recurrence. The patient-level recurrence result was considered positive when either local tumor progression or new intrahepatic recurrence was present. Because the primary objective was to evaluate overall patient-level diagnostic performance, the two recurrence patterns were included in the same primary endpoint and were not analyzed as separate diagnostic subgroups.

CEUS evaluation included a whole-liver grayscale survey and targeted qualitative and temporal contrast-enhanced assessment of the treated area and any additional suspicious intrahepatic lesion.

Qualitative parameters included enhancement patterns in the ablation zone during the arterial phase (10–30 seconds), portal venous phase (30–120 seconds), and delayed phase (120–360 seconds), categorized as no enhancement, homogeneous enhancement, heterogeneous enhancement, or peripheral (ring-like/nodular) enhancement. Additional features recorded were peritumoral enhancement (present/absent) and tumor margin characteristics (smooth/irregular).

Temporal parameters included time to initial enhancement, time to peak enhancement, and onset of washout, with documentation of washout phase (portal or delayed) and washout speed (rapid or slow).

MRI evaluation included whole-liver assessment of both qualitative and quantitative features, with particular attention to the treated-area margin and any newly developed lesion elsewhere in the liver.

Qualitative features included signal characteristics of the ablation zone on T2WI, DWI (b = 1000 s/mm²), and ADC maps. On dynamic contrast-enhanced sequences, the ablation zone was assessed during the arterial (25–30 seconds), portal venous (55–65 seconds), and delayed (2–3 minutes) phases for enhancement patterns (no enhancement, homogeneous enhancement, heterogeneous enhancement, peripheral ring/nodular enhancement, or internal nodular enhancement). The presence of peritumoral arterial phase hyperenhancement, capsule-like enhancement (complete/incomplete/absent), and margin regularity (regular/irregular) were also recorded.

Quantitatively, regions of interest (ROIs) avoiding necrotic or hemorrhagic areas were selected on the largest cross-section of the ablation zone to measure mean ADC values. Reference ROIs (200–300 mm²) were placed in adjacent normal liver parenchyma (avoiding vessels/ducts) to measure liver ADC, and the ADC ratio was calculated as:


ADC ratio = Mean ADC of ablation zone/Mean ADC of liver parenchyma

Image interpretation was performed independently by two senior radiologists, each with at least 7 years of experience. At the time of the index-test interpretations, both readers were blinded to the reference-standard outcomes, subsequent follow-up findings, and final clinical diagnoses. Each patient was evaluated using three reading strategies:

1. CEUS-only interpretation;
2. MRI-only interpretation using both qualitative and quantitative MRI findings; and
3. combined CEUS–MRI interpretation, in which the available findings from both modalities were reviewed together.

Separate binary classifications of early recurrence present or absent were recorded for each reading strategy. The combined CEUS–MRI strategy did not use a prespecified AND/OR rule, numerical scoring system, regression equation, probability estimate, or diagnostic threshold. It therefore represented expert integrated interpretation rather than a formal diagnostic model. The retrospective reading records did not document randomized ordering of the single-modality assessments or a formal washout interval between the CEUS-only, MRI-only, and combined assessments. Accordingly, the diagnostic performance of the combined strategy was considered exploratory and was interpreted cautiously.

If the two radiologists disagreed on the interpretation of any reading strategy, a third senior radiologist with more than 25 years of experience independently reviewed the case, and the resulting decision was used as the final classification for that reading strategy. The reference standard for recurrence was established separately from the CEUS-only, MRI-only, and combined CEUS–MRI classifications. Histopathological confirmation was used when available. For patients without pathological confirmation, recurrence status was determined using a composite reference standard based on longitudinal findings during the 12-month follow-up period, including interval lesion growth, persistent or progressive arterial-phase enhancement followed by washout on subsequent contrast-enhanced CT or MRI, changes in serum AFP when available, and the subsequent clinical course or treatment response. Reference-standard adjudication was performed independently by two hepatobiliary radiologists who had not participated in the original index-test interpretations, with disagreements resolved by consensus with a third senior radiologist. A positive finding on CEUS, MRI, or combined CEUS–MRI alone was not considered sufficient to establish recurrence.

### 2.7 Statistical analysis

Statistical analyses were performed using SPSS version 25.0. The analysis proceeded in three stages: comparison of baseline demographic and clinical characteristics between the early recurrence and no early recurrence groups, comparison of CEUS and MRI features between the two groups, and evaluation of the diagnostic performance and interobserver agreement of the three imaging strategies.

Continuous variables were summarized as mean ± standard deviation. The distribution and variance characteristics of continuous variables were assessed before between-group comparisons. Variables meeting the assumptions of approximate normality and homogeneity of variance were compared using the independent-samples t test; otherwise, the Mann–Whitney U test was used. Categorical variables were summarized as numbers and percentages and compared using the chi-square test. Fisher’s exact test was used when the expected frequency in any cell was less than 5. Effect estimates were reported as mean differences with 95% confidence intervals for continuous variables and as unadjusted odds ratios with 95% confidence intervals for binary categorical variables. Baseline demographic and clinical comparisons were considered secondary exploratory analyses. Because recurrence-risk modeling was not a prespecified objective of this diagnostic study, variables were not selected from univariable comparisons to construct a *post hoc* multivariable model, and no variable was interpreted as an independent risk factor.

For each imaging strategy, the final radiological assessment was recorded as a binary classification of early recurrence present or absent. Diagnostic performance was evaluated using 2 × 2 contingency tables based on the prespecified composite reference standard described above. True-positive, false-positive, true-negative, and false-negative results were used to calculate sensitivity [TP/(TP + FN)], specificity [TN/(TN + FP)], positive predictive value [TP/(TP + FP)], negative predictive value [TN/(TN + FN)], and overall accuracy [(TP + TN)/total number of patients]. Because no continuous patient-level diagnostic scores or multiple prespecified thresholds were generated for CEUS, MRI, or combined CEUS–MRI, ROC curves, AUC values, and DeLong comparisons were not included in the revised analysis.

Interobserver agreement between the two radiologists was evaluated separately for CEUS, MRI, and combined CEUS–MRI using Cohen’s kappa coefficient. A κ value >0.75 was interpreted as excellent agreement, 0.40–0.75 as moderate-to-good agreement, and<0.40 as poor agreement. Because the inclusion criteria required complete imaging and follow-up information, no missing-value imputation was performed. All statistical tests were two-sided, and P<0.05 was considered statistically significant.

## 3 Results

### 3.1 Baseline characteristics of patients

A total of 173 eligible patients with HCC were included in the analysis. All patients underwent RFA-TACE and completed the scheduled imaging examinations and standardized clinical follow-up. Early recurrence within 12 months after RFA-TACE occurred in 109 patients (63.01%), whereas 64 patients (36.99%) had no early recurrence during this period. The early recurrence endpoint included both local tumor progression at or adjacent to the treated area and new intrahepatic recurrence arising elsewhere in the liver; these recurrence patterns were not analyzed separately because the primary analysis focused on the overall diagnostic performance of the three imaging strategies. Final recurrence classification was based on patient-level whole-liver assessment, whereas persistent viable tumor at the initial 1-month examination was considered incomplete local treatment and was not included as a recurrence event. The baseline demographic and clinical characteristics of the two groups are presented in **Table 1**. In unadjusted baseline comparisons, BCLC stage C disease was associated with higher odds of early recurrence than BCLC stage B disease (OR = 4.10, 95% CI: 1.97–8.52; P<0.001). Child-Pugh class B liver function (OR = 3.31, 95% CI: 1.43–7.69; P = 0.004) and pretreatment AFP >100 ng/mL (OR = 2.02, 95% CI: 1.08–3.79; P = 0.026) were also associated with early recurrence. Tumor diameter >5 cm did not reach statistical significance (OR = 1.86, 95% CI: 0.97–3.57; P = 0.059). The mean age difference between the groups was 2.18 years (95% CI: −0.11 to 4.47; P = 0.062). Sex and HCC etiology were not significantly associated with early recurrence. These findings represent exploratory unadjusted associations and should not be interpreted as independent risk factors.

**Table 1:** Baseline demographic and clinical characteristics according to early recurrence status within 12 months after RFA-TACE.

Characteristic	Early recurrence group (n=109)	No early recurrence group (n=64)	t/χ²	P-value
Age, years	58.21 ± 7.45	56.03 ± 7.28	1.89	0.062
Sex	–	–	0.63	0.429
Male	92 (84.40%)	51 (79.69%)	–	–
Female	17 (15.60%)	13 (20.31%)	–	–
BCLC stage	–	–	15.34	<0.001
Stage B	56 (51.38%)	52 (81.25%)	–	–
Stage C	53 (48.62%)	12 (18.75%)	–	–
Child-Pugh class	–	–	8.30	0.004
Class A	74 (67.89%)	56 (87.50%)	–	–
Class B	35 (32.11%)	8 (12.50%)	–	–
Pretreatment AFP level	–	–	4.93	0.026
≤100 ng/mL	44 (40.37%)	37 (57.81%)	–	–
>100 ng/mL	65 (59.63%)	27 (42.19%)	–	–
Tumor diameter	–	–	3.58	0.059
≤5 cm	59 (54.13%)	44 (68.75%)	–	–
>5 cm	50 (45.87%)	20 (31.25%)	–	–
HCC etiology	–	–	0.22	0.897
HBV	80 (73.39%)	47 (73.44%)	–	–
HCV	15 (13.76%)	10 (15.63%)	–	–
Other causes	14 (12.84%)	7 (10.94%)	–	–

Data are presented as mean ± standard deviation or n (%). AFP, alpha-fetoprotein; BCLC, Barcelona Clinic Liver Cancer; HBV, hepatitis B virus; HCV, hepatitis C virus; RFA-TACE, radiofrequency ablation combined with transarterial chemoembolization.

### 3.2 Imaging features associated with early recurrence

CEUS and MRI findings differed significantly between the early recurrence and no early recurrence groups.

On CEUS, arterial-phase nodular or irregular enhancement within the treated area was observed in 78.9% (86/109) of patients in the early recurrence group and 9.4% (6/64) in the no early recurrence group (P<0.001). Early contrast washout during the portal venous or delayed phase occurred in 82.6% (90/109) and 6.3% (4/64) of patients, respectively (P<0.001). Peritumoral enhancement (35.8% vs 12.5%, P = 0.001) and irregular margins (63.3% vs 18.8%, P<0.001) were also more frequent in the early recurrence group (**Table 2**). The corresponding unadjusted effect estimates were OR = 36.14 (95% CI: 13.86–94.23) for arterial-phase nodular or irregular enhancement, OR = 71.05 (95% CI: 23.03–219.22) for early washout, OR = 3.90 (95% CI: 1.69–9.02) for peritumoral enhancement, and OR = 7.48 (95% CI: 3.57–15.65) for irregular margins.

**Table 2:** CEUS findings according to early recurrence status within 12 months after RFA-TACE.

CEUS imaging feature	Early recurrence group (n=109)	No early recurrence group (n=64)	χ²	P-value
Arterial phase nodular/irregular enhancement	86 (78.9%)	6 (9.4%)	78.28	<0.001
Early washout (portal/delayed phase)	90 (82.6%)	4 (6.3%)	94.66	<0.001
Peritumoral enhancement	39 (35.8%)	8 (12.5%)	11.04	0.001
Irregular margins	69 (63.3%)	12 (18.8%)	32.15	<0.001

Data are presented as n (%). CEUS, contrast-enhanced ultrasound; RFA-TACE, radiofrequency ablation combined with transarterial chemoembolization.

On MRI, DWI hyperintensity was observed in 91.7% (100/109) of patients in the early recurrence group and 25.0% (16/64) in the no early recurrence group (P<0.001). Arterial-phase nodular or irregular enhancement (85.3% vs 10.9%), delayed-phase washout (79.8% vs 7.8%), peritumoral arterial enhancement (42.2% vs 15.6%), and irregular margins (70.6% vs 17.2%) were also more frequent in the early recurrence group (all P<0.001). The mean ADC value was lower in the early recurrence group than in the no early recurrence group (1.05 ± 0.18 vs 1.32 ± 0.15 ×10^-3^ mm²/s, P<0.001), as was the ADC ratio (0.89 ± 0.12 vs 1.15 ± 0.14, P<0.001) (**Table 3**). The corresponding unadjusted odds ratios were 33.33 (95% CI: 13.74–80.87) for DWI hyperintensity, 47.33 (95% CI: 18.35–122.07) for arterial-phase nodular or irregular enhancement, 46.66 (95% CI: 16.73–130.16) for delayed-phase washout, 3.94 (95% CI: 1.82–8.55) for peritumoral arterial enhancement, and 11.59 (95% CI: 5.37–25.02) for irregular margins. The between-group mean difference was −0.27 ×10^-3^ mm²/s for the mean ADC value (95% CI: −0.32 to −0.22) and −0.26 for the ADC ratio (95% CI: −0.30 to −0.22).

**Table 3:** MRI findings according to early recurrence status within 12 months after RFA-TACE.

MRI imaging feature	Early recurrence group (n=109)	No early recurrence group (n=64)	t/χ²	P-value
Marked hyperintensity on DWI	100 (91.7%)	16 (25.0%)	81.31	<0.001
Arterial phase nodular/irregular enhancement	93 (85.3%)	7 (10.9%)	91.47	<0.001
Delayed phase washout	87 (79.8%)	5 (7.8%)	83.96	<0.001
Peritumoral arterial enhancement	46 (42.2%)	10 (15.6%)	13.01	<0.001
Irregular margins	77 (70.6%)	11 (17.2%)	46.10	<0.001
Mean ADC value (×10^-3^ mm²/s)	1.05 ± 0.18	1.32 ± 0.15	-10.60	<0.001
ADC ratio	0.89 ± 0.12	1.15 ± 0.14	-12.42	<0.001

Data are presented as n (%) or mean ± standard deviation. ADC, apparent diffusion coefficient; DWI, diffusion-weighted imaging; MRI, magnetic resonance imaging; RFA-TACE, radiofrequency ablation combined with transarterial chemoembolization.

### 3.3 Diagnostic performance of CEUS, MRI, and exploratory combined CEUS–MRI interpretation

Using the prespecified composite reference standard, diagnostic performance was calculated for CEUS-only interpretation, MRI-only interpretation, and exploratory combined CEUS–MRI interpretation using 2 × 2 contingency tables (**Table 4**). For CEUS, sensitivity was 80.7% (95% CI: 72.3%–87.0%), specificity was 82.8% (95% CI: 71.8%–90.1%), positive predictive value was 88.9% (95% CI: 81.2%–93.7%), negative predictive value was 71.6% (95% CI: 60.5%–80.6%), and accuracy was 81.5% (95% CI: 75.1%–86.6%). For MRI, sensitivity was 88.1% (95% CI: 80.7%–92.9%), specificity was 87.5% (95% CI: 77.2%–93.5%), positive predictive value was 92.3% (95% CI: 85.6%–96.1%), negative predictive value was 81.2% (95% CI: 70.4%–88.6%), and accuracy was 87.9% (95% CI: 82.2%–91.9%). For combined CEUS–MRI, sensitivity was 92.7% (95% CI: 86.2%–96.2%), specificity was 93.8% (95% CI: 85.0%–97.5%), positive predictive value was 96.2% (95% CI: 90.6%–98.5%), negative predictive value was 88.2% (95% CI: 78.5%–93.9%), and accuracy was 93.1% (95% CI: 88.3%–96.0%). Compared with MRI alone, combined CEUS–MRI yielded five additional true-positive classifications and four additional true-negative classifications, corresponding to nine additional correct classifications among the 173 patients. Interobserver agreement was good for all three assessment strategies (CEUS, κ=0.82; MRI, κ=0.85; combined CEUS–MRI, κ=0.78). Because the combined strategy was based on expert integrated interpretation without a prespecified combination algorithm and because randomized reading order and a formal washout interval were not documented, its apparently higher diagnostic performance should be regarded as exploratory.

**Table 4:** Diagnostic performance of CEUS, MRI, and exploratory combined CEUS–MRI interpretation for detecting early recurrence within 12 months after RFA-TACE.

Diagnostic method	Sensitivity, % (n/N)	Specificity, % (n/N)	PPV, % (n/N)	NPV, % (n/N)	Accuracy, % (n/N)
CEUS	80.7 (88/109)	82.8 (53/64)	88.9 (88/99)	71.6 (53/74)	81.5 (141/173)
MRI	88.1 (96/109)	87.5 (56/64)	92.3 (96/104)	81.2 (56/69)	87.9 (152/173)
Exploratory combined CEUS–MRI interpretation	92.7 (101/109)	93.8 (60/64)	96.2 (101/105)	88.2 (60/68)	93.1 (161/173)

Data are presented as percentage (numerator/denominator). CEUS, contrast-enhanced ultrasound; MRI, magnetic resonance imaging; NPV, negative predictive value; PPV, positive predictive value; RFA-TACE, radiofrequency ablation combined with transarterial chemoembolization.

### 3.4 Representative imaging findings in a patient with early recurrence after RFA-TACE

**Figure 1:**
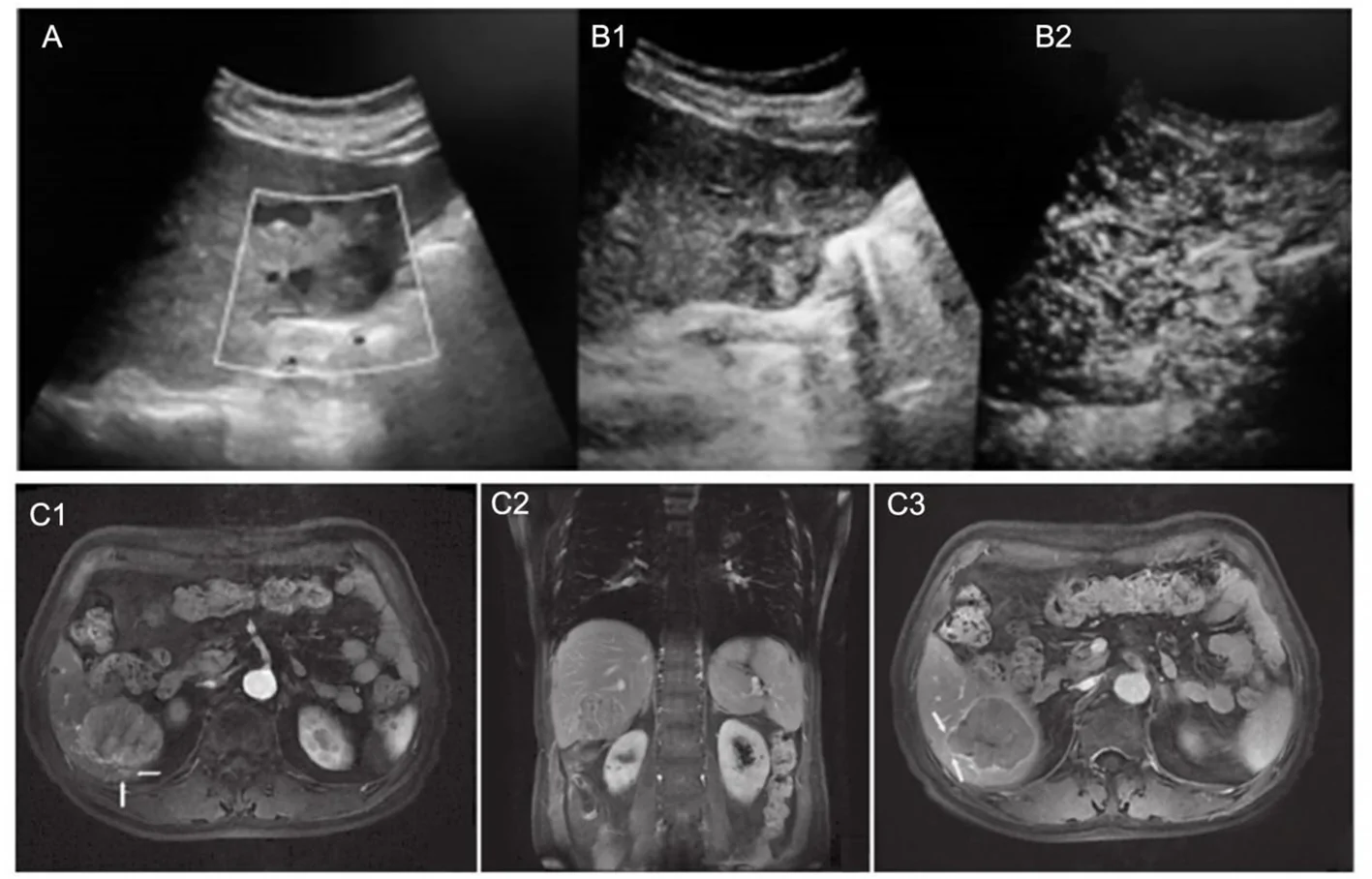
Representative conventional ultrasound, CEUS, and contrast-enhanced MRI findings in a 46-year-old man with HCC recurrence after RFA-TACE. **(A)** Conventional ultrasound shows blood-flow signals at the margin of the treated area. **(B1)** Arterial-phase CEUS at 18 seconds shows irregular peripheral enhancement and peritumoral radial enhancement. **(B2)** Portal venous-phase CEUS at 45 seconds shows contrast washout and hypoenhancement of the lesion. **(C1)** Arterial-phase MRI at 28 seconds shows peritumoral enhancement surrounding a lesion measuring approximately 4.3 cm. **(C2)** Portal venous-phase MRI at 60 seconds shows lower lesion enhancement than the adjacent liver parenchyma. **(C3)** Delayed-phase MRI at 180 seconds shows further contrast washout, an irregular lesion margin, and capsule-like enhancement. CEUS, contrast-enhanced ultrasound; HCC, hepatocellular carcinoma; MRI, magnetic resonance imaging; RFA-TACE, radiofrequency ablation combined with transarterial chemoembolization.

**Figure 1** presents representative conventional ultrasound, CEUS, and contrast-enhanced MRI findings in a 46-year-old man with HCC recurrence detected three months after RFA-TACE. Conventional ultrasound demonstrated blood-flow signals at the margin of the treated area. CEUS showed irregular peripheral enhancement during the arterial phase and subsequent contrast washout during the portal venous phase. Contrast-enhanced MRI demonstrated peritumoral arterial enhancement, reduced enhancement during the portal venous phase, and further washout with an irregular margin during the delayed phase. HCC recurrence was subsequently confirmed by biopsy.

## 4 Discussion

This study retrospectively analyzed 173 patients with HCC treated with RFA-TACE and compared the descriptive diagnostic performance of CEUS, MRI, and combined CEUS–MRI for identifying early recurrence. Exploratory combined CEUS–MRI interpretation showed higher descriptive sensitivity, specificity, and accuracy than either modality alone. The observed 63.01% early recurrence proportion should be interpreted as a cohort-specific finding in patients with BCLC stage B or C disease who underwent RFA-TACE, rather than as a general estimate of the contemporary 12-month recurrence risk for all patients with HCC. The observed diagnostic pattern may be explained by the complementary information provided by CEUS and MRI. Previous studies have shown that CEUS can provide diagnostic information comparable or complementary to MRI for the characterization of HCC observations (15, 16). Arterial-phase hyperenhancement and contrast washout are major imaging features associated with HCC across CEUS and CT/MRI assessments (17), and CEUS has demonstrated favorable diagnostic performance when evaluated alongside corresponding MRI findings (18). In the present cohort, CEUS provided real-time information on vascular enhancement and contrast washout, whereas MRI provided whole-liver anatomical and diffusion-related information. This complementary information may explain the higher descriptive diagnostic performance observed with exploratory combined CEUS–MRI interpretation.

Current Japanese and international guidance emphasizes individualized HCC management and the appropriate selection of imaging and locoregional treatment strategies (19, 20). Previous studies have evaluated hepatic resection combined with intraoperative radiofrequency ablation (21), fusion imaging and cone-beam computed tomography-guided radiofrequency ablation for HCC poorly visualized on ultrasonography (22), CT/MR–ultrasound fusion-assisted thermal ablation (23), feeding-vessel ablation for tumors in challenging anatomical locations (24), fusion-assisted thermal ablation for medium-sized HCC (25), FAPI PET/CT for suspected recurrent HCC (26), and ultrasound-guided microwave ablation for caudate-lobe HCC (27). These studies provide relevant clinical, diagnostic, and technical context but do not directly validate the exploratory combined CEUS–MRI interpretation strategy used in the present study. The present study specifically evaluated CEUS, dynamic contrast-enhanced MRI, and quantitative DWI/ADC parameters within the same post-RFA-TACE cohort. Nevertheless, direct numerical comparisons across studies should be interpreted cautiously because of differences in treatment setting, patient population, recurrence definition, imaging criteria, unit of analysis, image-reading procedures, and reference standards.

The principal contribution of this study is the evaluation of CEUS perfusion characteristics, dynamic MRI findings, and quantitative DWI/ADC parameters within the same cohort during early follow-up after RFA-TACE. The potential clinical value of exploratory combined interpretation may lie in its use as a complementary problem-solving approach when a single modality is technically limited or produces indeterminate findings. However, because the combined strategy was based on expert integrated judgment rather than a prespecified and independently validated algorithm, its apparently higher diagnostic performance should not be interpreted as evidence of a definitive or reproducible diagnostic model. Compared with MRI alone, combined CEUS–MRI increased sensitivity by 4.6 percentage points, specificity by 6.3 percentage points, and accuracy by 5.2 percentage points, corresponding to nine additional correct classifications in this cohort. This incremental diagnostic gain may be clinically relevant when an indeterminate or technically limited MRI examination could delay further evaluation or lead to an unnecessary intervention. However, the present study did not determine whether these additional correct classifications changed subsequent treatment decisions, shortened the time to retreatment, or improved progression-free or overall survival. Direct examination costs were also not collected, and no formal cost-effectiveness analysis was performed. Therefore, the present findings support selective use of CEUS as a complementary examination when MRI findings are equivocal or technically limited, rather than routine dual-modality imaging for every patient. In unadjusted exploratory analyses, BCLC stage C disease, Child-Pugh class B liver function, and pretreatment AFP >100 ng/mL were associated with early recurrence, whereas tumor diameter >5 cm did not reach statistical significance. Because no prespecified multivariable recurrence-risk model was developed, these findings should not be interpreted as independent risk factors or as validated criteria for selecting patients for dual-modality surveillance. The optimal target population, management impact, survival benefit, and cost-effectiveness require prospective evaluation.

Several limitations should be acknowledged. First, this was a retrospective study conducted at a single center in China, and most patients had HBV-related HCC. Therefore, selection bias cannot be excluded, and the findings may not be directly generalizable to populations with different ethnic backgrounds, viral-hepatitis prevalence, HCC etiologies, disease stages, or treatment pathways. Previous commentary has similarly emphasized that findings from Chinese HCC cohorts should be extrapolated cautiously to non-HBV- or non-HCV-related populations (28). Second, the combined CEUS–MRI strategy was based on expert integrated interpretation without a prespecified AND/OR rule, numerical scoring system, regression equation, or diagnostic threshold. In addition, the retrospective reading records did not document randomized reading order or a formal washout interval between the single-modality and combined assessments. Readers may therefore have remembered their earlier interpretations when performing the combined assessment, introducing recall and review bias and potentially inflating the apparent performance of the combined strategy. The combined results should consequently be considered exploratory until they are validated prospectively using a prespecified combination rule, randomized reading order, an adequate washout interval, and independent readers. Third, histopathological confirmation was not available for all patients, and the composite reference standard therefore relied partly on longitudinal imaging and clinical follow-up. Although reference-standard adjudication was performed independently and a positive index examination alone was not considered sufficient to establish recurrence, partial incorporation bias and verification bias cannot be completely excluded. Consequently, the reported diagnostic performance may have been overestimated and should be interpreted cautiously. Although the final patient-level diagnosis was based on whole-liver assessment, the predefined feature-level comparisons focused primarily on the treated area and its margins. In addition, local tumor progression and new intrahepatic recurrence were included in the same early recurrence endpoint and were not evaluated separately. Therefore, the relative diagnostic performance of CEUS and MRI for different spatial recurrence patterns could not be determined. Finally, the study did not collect direct examination costs or evaluate downstream changes in clinical management, time to retreatment, progression-free survival, or overall survival. Consequently, the present data cannot establish the cost-effectiveness of dual-modality imaging or demonstrate that its incremental diagnostic yield translates into improved survival outcomes. Prospective comparative studies incorporating health-economic evaluation, treatment-decision outcomes, and long-term patient outcomes are required to determine the incremental clinical value of combined CEUS–MRI.

## 5 Conclusion

This study showed that exploratory combined CEUS–MRI interpretation had higher descriptive sensitivity, specificity, and accuracy than either CEUS-only or MRI-only interpretation for identifying early recurrence after RFA-TACE. The principal advantage of combined interpretation lies in integrating CEUS assessment of vascular perfusion and contrast washout with MRI evaluation of vascular enhancement, tissue structure, and water-molecule diffusion. Given its incremental diagnostic gain over MRI alone and the absence of direct cost and outcome data, exploratory combined CEUS–MRI interpretation should be considered selectively when findings from a single modality are technically limited or indeterminate, rather than being routinely applied to all patients. Prospective multicenter studies using a prespecified combination rule, independent readers, randomized reading order, and an adequate washout interval are required to determine its effects on clinical management, long-term outcomes, and cost-effectiveness before routine implementation.

## Data Availability

The original contributions presented in the study are included in the article/supplementary material. Further inquiries can be directed to the corresponding author.
